# On Persons, Populations, and Causal Mechanisms Some Critical Reflections on Lundh (2023)

**DOI:** 10.17505/jpor.2024.26272

**Published:** 2024-05-23

**Authors:** James T. Lamiell

**Affiliations:** Psychology Department, Georgetown University, Washington D.C., USA

**Keywords:** knowledge objectives, causation, persons, critical personalism

## Abstract

While agreeing with Lundh (2023) on many of his major points, this article also questions the notion of a ‘population psychology.’ It is argued that the knowledge produced in population-level studies, whether correlational, experimental, or mixed, is inherently demographic in nature. Concerning individual-level studies, agreement with Lundh (2023) is expressed concerning the need to distinguish between a conception of individuals as mere depositories of neurophysiological mechanisms on the one hand, and as active, purposeful agents on the other. It is suggested that the conceptual framework called ‘critical personalism’ would well serve a scientific psychology committed to the latter view.

I am grateful to Professor Lundh for the invitation to author a commentary on his article ‘Person, Population, Mechanism: Three Main Branches of Psychological Science’ (Lundh, 2023). I fully agree with him concerning the need for much more conceptual clarity than currently exists within the mainstream of psychology with respect to the issues he addresses. I intend the historical and philosophical reflections shared in this commentary to contribute further to that same end.

## On the Problematic Assumption of Epistemic Continuity in the Historic Ascendance of ‘Population Psychology’

The experimental psychology that Wilhelm Wundt (1832-1920) is credited with launching in the German city of Leipzig in 1879 was, as Lundh noted, an *individual* psychology. More precisely, it was an experimental discipline aimed at discovering the general laws presumed to regulate the ‘psychological doings’—phenomena such as sensations, perceptions, judgments, emotions, recollections, cognitions, behaviors, development—of individual human beings. Under the logic of the laboratory investigations conducted within that discipline, experimental findings were fully defined by the results obtained with each individual investigated, and the generality of the findings would depend upon their commonality across investigated individuals (cf. Danziger, 1990; Lamiell, 2019).^1^

Early in the 20th century, however, the original Wundt-ian model of experimentation was gradually abandoned in favor of a *treatment group* model (Danziger, 1990). Unlike the logic of the original, the logic of the treatment group model was no longer such that experimental findings were fully defined by the results obtained with any individual subject. Instead, the findings remained indeterminate until, for *all* of the individuals targeted for investigation—the more the better—results had been obtained, aggregated, and statistically analyzed. In the prosecution of this model, research psychologists embraced population-level statistical methods of investigation that had had no place in Wundt-ian style experimentation, but were taken up in a newer sub-discipline devoted to the study of individual differences within populations in various psychological doings. That sub-discipline was one that William Stern (1871-1938) founded in 1900 (Stern, 1900) and proposed to call ‘differential psychology.’^2^

Despite the epistemically radical difference between the Wundt-ian and treatment group models of experimentation, the belief was broadly embraced at the time (and continues to prevail today) that the treatment group model offers *an alternative means to the same overall knowledge objective* as the original Wundt-ian model, i.e., the objective of advancing our scientific understanding of the psychological doings of individuals. Elsewhere, I have referred to that notion as *the assumption of epistemic continuity* in the disciplinary migration from Wundt-ian style experimentation to treatment group experimentation (cf. Lamiell, 2019). David N. Bakan (1921-2004) was one prominent 20th century psychologist who, already by mid-century, was calling attention to the mistakenness of that assumption (Bakan, 1955, 1966). A dozen or so years later, Fred N. Kerlinger (1910-1991) explicitly identified—but did not propose a solution for—what he termed the 'troublesome paradox' within mainstream psychology created by studying groups of individuals when the theoretical objective was to advance our understanding of individuals (Kerlinger, 1979). Danziger (1987) would later explain that the appearance of a solution to that paradox was achieved *de facto* by the broad acceptance within the psychological community of the view that “the statistical structure of the data based on the responses of many individuals (may be) assumed to conform to the structure of the relevant psychological processes operating on the individual level” (Danziger, 1987, p. 45, parentheses added).

In the above-cited work (Lamiell, 2019), not cited by Lundh (2023), I have unpacked for closer critical examination the way of thinking articulated by Danziger (1987), and I have explained just why that way of thinking will not hold up to close critical scrutiny. As jarring as it may be to the sensibilities of many contemporary psychologists, the bottom line is that knowledge of populations—whether the knowledge is correlational or experimental, and whether the populations are extant and widely recognized populations or those just theoretically envisioned in the creation of experimental treatment groups—is actually and quite literally knowledge of no one. Contrary to the assumption of epistemic continuity, but as the 19th century German philosopher Moritz Wilhelm Drobisch (1802-1896) wrote in 1867:

It is only through a great failure of understanding (that) the mathematical fiction of an average (aggregate) man … (can) be elaborated as if all individuals … possess a real part of whatever obtains for this average (aggregate) person (quoted in Porter, 1986, p. 171, parentheses added).

Note that this does not preclude the possibility of discovering what Lundh (2023) discusses as “group-to-individual generalizability” (p. 79). It does mean that any such discovery will *always* require the investigation of individual cases considered as such, precisely *because* no group-level reality can ever be validly assumed to capture the reality of any given individual case. But now: if methods for establishing individual-level realities exist (they do), and if knowledge of individual-level realities is, as it was at scientific psychology’s founding, the discipline’s ultimate knowledge objective, then just how the effort to realize that objective is ever served by population-level studies—whether or not they are followed up with attempts to determine population-to-individual generalizability—is not apparent.

The conceptual waters are only further muddied when it is suggested that “*'causal structures*” involving psychological phenomena can be “operative at the … population level” (Lundh, 2023, p. 80). As Harré (1981) noted, “causal processes occur only in individual beings, since mechanisms of action, *even when we act as members of collectives*, must be realized in particular persons” (p. 14, emphasis added). At the very least, I think it incumbent on Lundh to clarify just how, for example, “affect intensity as a trait variable,” i.e., a variable defined by individual *differences* in affect intensity, can function *causally* at the *population* level. Equally dubious, and for the same reason, is the notion of “population-level effects of various psychological interventions” (p. 75), for where we speak as scientists of ’effects’ we have necessarily implied the workings of *causes*, and Harré's (1981) just-quoted stipulation applies here, too.

In light of the foregoing considerations, I stand by my contention that the knowledge generated by population-level studies is fundamentally and irreducibly demographic in nature (cf. Lamiell, 2019), and so I must respectfully disagree with Lundh's (2023) suggestion that that contention “partly misses the mark” (p. 80).

## On the Importance of Viewing Individuals as Persons in ‘Mechanism Psychology’

The need stipulated by Harré (1981) for knowledge of mechanisms of action as they transpire in individual beings is presumably one that could in principle be met by work in the sub-discipline of neurophysiological psychology. Regarding Lundh's (2023) discussion of current work in this domain, I fully endorse the wariness he expresses of conceptions of individuals as mere ‘depositories of diverse mechanisms’ instead of as ‘active, purposeful agents.’ Conceptions of the former sort effectively reduce persons to things, and while Lundh draws particular attention to the inadequacy of such a reduction for understanding the dynamics of patient-therapist relationships in psychotherapy, the basic point applies to inter-personal relationships more broadly.^3^

Importantly, Lundh's discussion of ‘mechanism psychology’ helps to make clear the fact that a philosophically and theoretically viable conception of *persons* is not, *ipso facto*, achieved simply by studying individual organisms. That much is necessary, to be sure. More than that, however, what is required is a coherent conceptual framework within which to distinguish mere ‘depositories of neurophysiological mechanisms’ from ‘active, purposeful agents.’ The system of thought that the aforementioned William Stern called critical personalism is one such framework, and its very basis is the distinction between persons and things. In recent publications (e.g., Lamiell, 2021, Lamiell, 2024), I have extended my long-running effort to introduce contemporary thinkers unfamiliar with Stern's work to his ideas and their potential both for reviving psychological science and for grounding a socio-cultural ethos. The ideas I discuss in those works, though not consonant with all of the ideas presented by Lundh (2023), nicely complement his views in most respects, and reinforce the importance of the issues he has raised in his highly commendable article.

## Footnotes

1 This model is essentially the one that Lundh (2023) describes Hayes and colleagues as advocating for their research program investigating the processes of change and development in individuals.

2 An English translation of Stern’s seminal 1900 book (Stern, 1900) has recently been completed by Serge Nicolas (b. 1962) and the present author (b. 1950) and will be published soon by Palgrave-Macmillan.

3 Also, and altogether appropriately, Lundh (2023) takes note of the conceptual hazard in mechanism-level research of a fallacy re-ferred to by M. R. Bennett (a philosopher born in 1939) and P. M. S. Hacker (a neuroscientist also born in 1939) as the ‘mereological fallacy’: the fallacy of attributing to parts of per-sons’ physical beings, such as their brains or some part(s) of their brains, psychological doings (e.g., perceiving, judging, emoting) that can sensibly be attributed only to whole persons.

## Action editor

Lars R. Bergman served as action editor for this article.

## Open access

This article is distributed under the terms of the Creative Commons Attribution 4.0 International License (http://creativecommons.org/licenses/by/4.0/), which permits unrestricted use, distribution, and reproduction in any medium, provided you give appropriate credit to the original author(s) and the source, provide a link to the Creative Commons license, and indicate if changes were made.

## References

[cit0001] Bakan, D. (1955). The general and the aggregate: A methodological distinction. *Perceptual and Motor Skills*, 5, 211-212. 10.2466/PMS.5.7.211-212

[cit0002] Bakan, D. (1966). The test of significance in psychological research. *Psychological Bulletin*, 66, 423-437. 10.1037/h0020412 5974619

[cit0003] Danziger, K. (1987). Statistical method and the historical development of research practice in American psychology. In L. Krueger, G. Gigerenzer, & M. S. Morgan (Eds.), *The probabilistic revolution, Vol. 2: Ideas in the sciences*, pp, 55-47. Cambridge, MA: MIT Press.

[cit0004] Danziger, K. (1990). *Constructing the subject: Historical origins of psychological research*. Cambridge, UK: Cambridge University Press.

[cit0005] Harré, R. (1981). The positivist-empiricist approach and its alternative. In P. Reason & J. Rowan (Eds.), *Human inquiry: A sourcebook of new paradigm research*, pp. 3-17). New York: Wiley.

[cit0006] Kerlinger, F. N. (1979). *Behavioral research: A conceptual approach*. New York: Holt, Rinehart, & Winston.

[cit0007] Lamiell, J. T. (2019). *Psychology's misuse of statistics and persistent dismissal of its critics*. London, UK: Palgrave-Macmillan. https://doi-org.ludwig.lub.lu.se/10.1007/978-3-030-12131-0

[cit0008] Lamiell, J. T. (2021). *Uncovering critical personalism: Readings from William Stern's contributions to scientific psychology*. London, UK: Palgrave-Macmillan.

[cit0009] Lamiell, J. T. (2024). *Primer in critical personalism: A framework for reviving psychological inquiry and for grounding a sociocultural ethos*. London: Routledge.

[cit0010] Lundh, L. G. (2023). Person, population, mechanism. Three main branches of psychological science. *Journal for Person-Oriented Research*, 9, 75-92. 10.17505/jpor.2023.25814. 38107200 PMC10722369

[cit0011] Porter, T. T. (1986). *The rise of statistical thinking: 1820-1900*. Princeton, NJ: Princeton University Press.

[cit0012] Stern, W. (1900). *Über Psychologie der individuellen Differenzen. (Ideen zu einer 'differentiellen Psychologie' (On the psychology of individual differences (Toward a 'differential psychology')*. Leipzig: Barth.

